# Supplementary material for: Proton enhanced dynamic battery chemistry for aprotic lithium–oxygen batteries
Source: Nat Commun. 2017 Feb 6;8:14308. doi: 10.1038/ncomms14308 (PMC5303818; doi:10.1038/ncomms14308)
Supplement: Supplementary Information — Supplementary Figures 1-16, Supplementary Tables 1-2 and Supplementary References [file ncomms14308-s1.pdf]

Supplementary Information

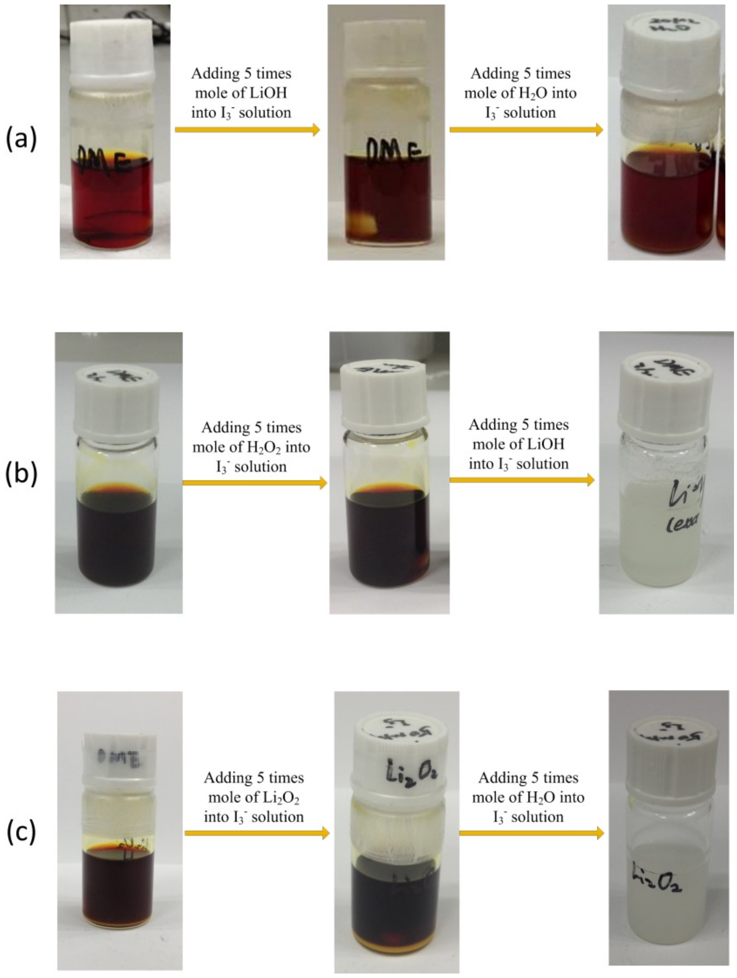

Supplementary Figure 1. Photographs show the titration experiments by dropwise adding ~5 times number of moles of (a) LiOH and LiOH+H_2O, (b) H_2O_2 and H_2O_2+LiOH, (c) Li_2O_2 and Li_2O_2+H_2O into the I_3^- solution in DME. The I_3^- solution was prepared by mixing 0.25 mmole I_2 and 0.25 mmole LiI in 5 mL DME solvent.

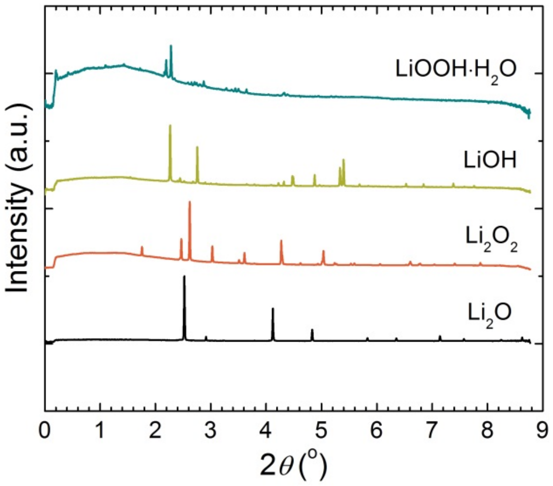

Supplementary Figure 2. The synchrotron X-ray diffraction patterns of Li_2O, Li_2O_2, LiOH, and $LiOOH \cdot H_2O$. The wavelength of X-ray source is 0.117418 Å. The X-ray diffraction pattern of LiOOH is clearly different from any other Li compounds tested here. In addition, the sharp peaks indicate the good crystallinity of LiOOH. The high-resolution XRD pattern of the presumed $LiOOH \cdot H_2O$ was also measured (Figure 1b). Based on the 28 primary peaks located between 3° to 16° (2 theta angle), the pattern could be well indexed using **DICVOL06** with a small zero-shift. A subsequent LeBail fit confirmed the indexing by achieving R_p: 5.66%, R_{wp}: 7.13%, R_{exp}: 4.20%, Chi^2: 2.88, i.e. the difference between the observed data and a the fitted lattice is very small. More precise lattice parameters and more detailed microstructural information of the powder samples could be derived from a Lebail fit of the high resolution XRD pattern below 2 Theta =22°. The results are **a= 6.36647 Å, b= 6.08798 Å, c= 3.20656 Å, α=79.59^o, β= 101.86^o, γ= 102.31^o, volume= 117.61 Å^3**, which is very close to the fitting result derived from the lower two theta angle.

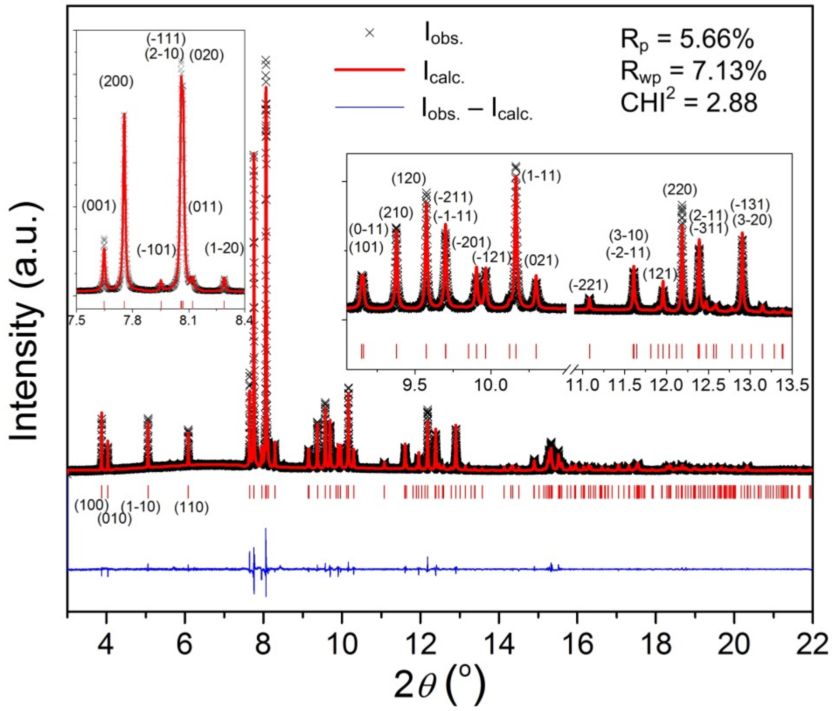

Supplementary Figure 3. LeBail Fitting of the high-resolution X-ray diffraction pattern of the chemically synthesized phase LiOOH·H_2O. The wavelength is λ = 0.41423±0.00004 Å.

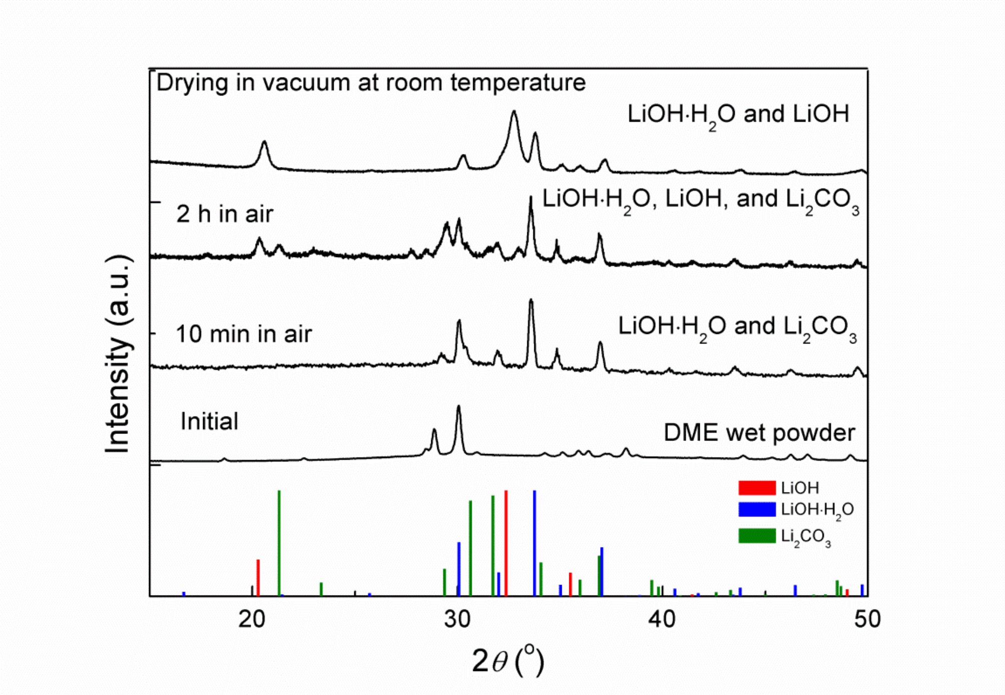

Supplementary Figure 4. XRD patterns of $LiOOH{\cdot}H_2O$ after exposed in air or under vacuum condition for different durations. The freshly made wet powder of $LiOOH{\cdot}H_2O$ was tested firstly. Then, the powder was left in air for 10 min and 2 hours, and tested again to monitor its stability. The formations of $LiOH{\cdot}H_2O$, LiOH, and Li_2CO_3 are clearly seen as time evolves, of which Li_2CO_3 could be a reaction product of $LiOOH{\cdot}H_2O$ or LiOH with CO_2 from air. In order to rule out the influence of CO_2, we dried $LiOOH{\cdot}H_2O$ in vacuum condition and did another XRD measurement, for which the main products become LiOH and $LiOH{\cdot}H_2O$, validating the above assumption.

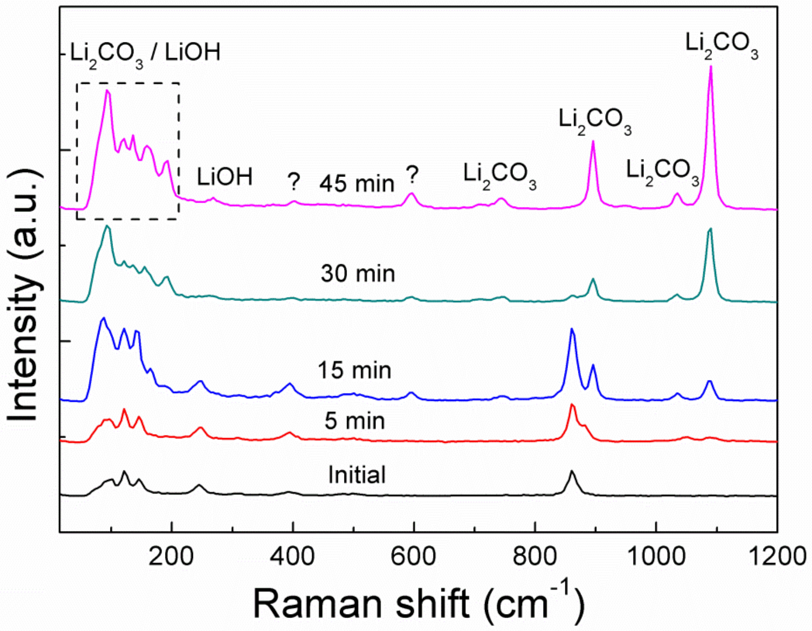

Supplementary Figure 5. Raman spectra of LiOOH·H_2O exposed in air for different durations. The LiOOH·H_2O was initially in the form of wet paste. The Raman spectra of LiOOH·H_2O evolved gradually and the characteristic peaks of LiOH and Li_2CO_3 become obvious even after 5 min exposure.[1, 2] The LiOOH was entirely converted to LiOH and Li_2CO_3 after 45 min, indicating LiOOH·H_2O is not stable in air. This result is consistent with the XRD measurements.

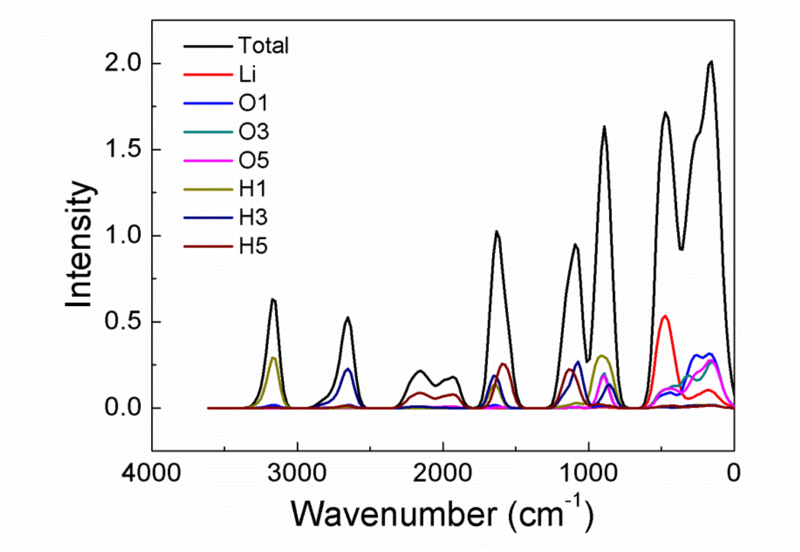

Supplementary Figure 6. Total (black) and partial (colored) phonon density of state for crystalline LiOOH·H_2O from DFT calculations. For the partial phonon DOS contributions relating to chemically equivalent atoms are added up. It has been confirmed by visualization that the peak calculated at 894 cm^{-1} peak corresponds to O-O stretching in the OOH^- group (superimposed by a H_2O rocking mode at 874 cm^{-1}), while the peak at 1643 cm^{-1} peak corresponds to H-O-H bending.

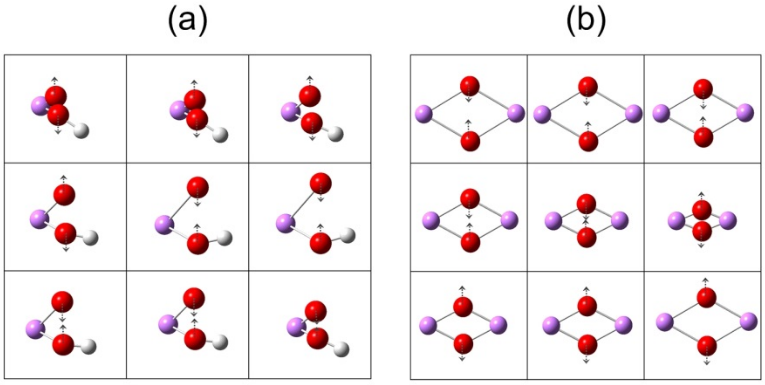

Supplementary Figure 7. Snapshots of the vibrations at (a) 838 cm^{-1} for LiOOH molecules, and (b) 828 cm^{-1} for Li_2O_2 molecules based on normal mode analysis. Both molecules were calculated at B3LYP/aug-cc-pVQZ level. LiOOH shows a strong Raman peak at 838 cm^{-1} which was in good accordance with the experimental data of 860 cm^{-1}. Based on the normal-mode analysis, this peak is assigned to be the O-O bond stretching. Similarly, Li_2O_2 demonstrated a Raman active O-O bond stretching at 828 cm^{-1} while the experiment value is at 790 cm^{-1}. Note that calculations presented in (a and b) are based on isolated molecules. In any case both these simplifications and the limited level of the DFT calculations may account for the error between the calculation and experimental data.

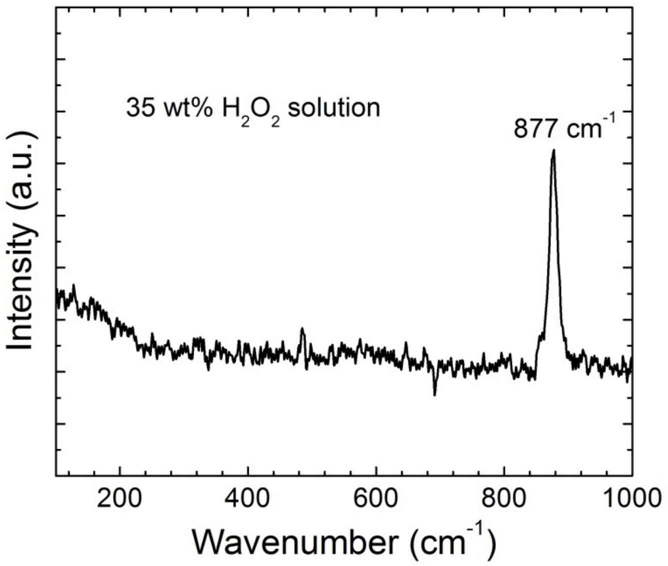

Supplementary Figure 8. Raman spectrum of H_2O_2 solution (35 *wt.*% in H_2O). The substrate is a piece of glass plate. In the measurement, H_2O_2 solution was dropped onto the glass substrate. A strong peak at around 877 cm^{-1} was observed, which is assigned to the O-O bond stretching of H_2O_2. In comparison, the O-O bond stretching of $LiOOH{\cdot}H_2O$ is located at around 860 cm^{-1}, distinct from that of H_2O_2.

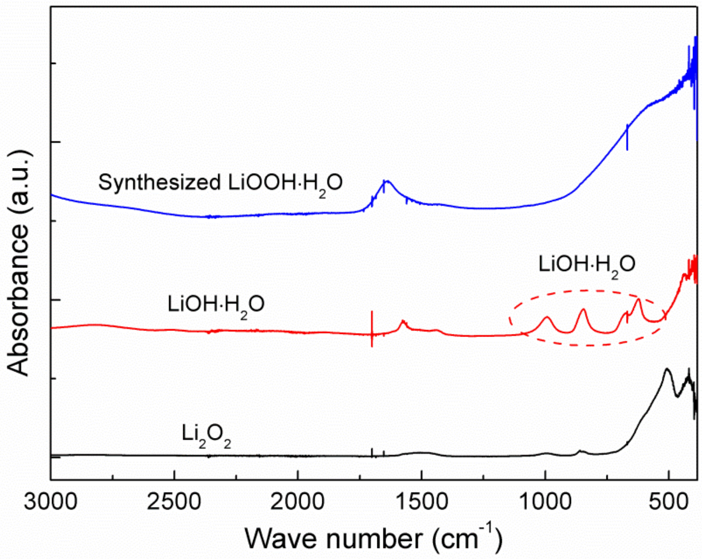

Supplementary Figure 9. (a) ATR-FTIR spectra of Li_2O_2, LiOH·H_2O, LiOOH·H_2O. Li_2O_2 presents characteristic peaks at around 524 and 432 cm^{-1}.[3] LiOH·H_2O exhibits a series of peaks from ~1000 to 610 cm^{-1}.[4] The FTIR spectrum of LiOOH·H_2O shows a characteristic peak at around 1642 cm^{-1}. LiOOH·H_2O was extracted from DME/H_2O solution.

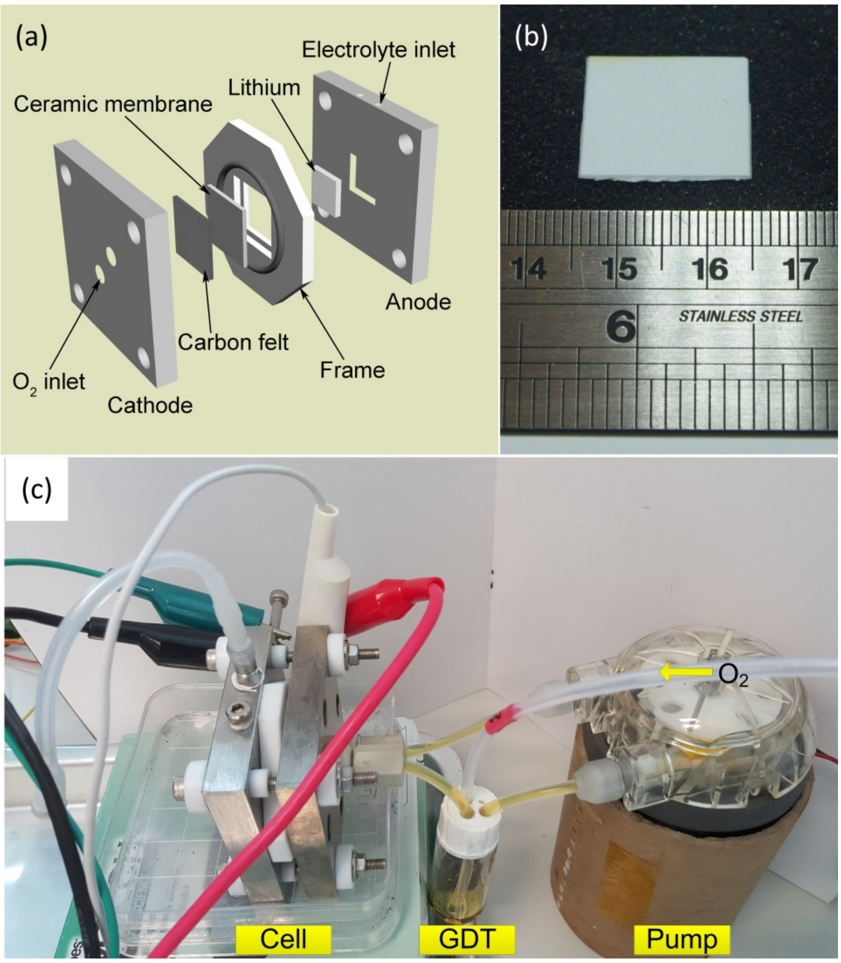

Supplementary Figure 10. (a) The exploded structure of static Li-O_2 battery used in this work. (b) The photograph of a piece of LAGP ceramic membrane. (c) The photograph of a redox flow lithium oxygen battery (RFLOB), in which the same battery stack was used for static cells except that the catholyte inlet/outlet on the cathode were converted into O_2 inlet/outlet.

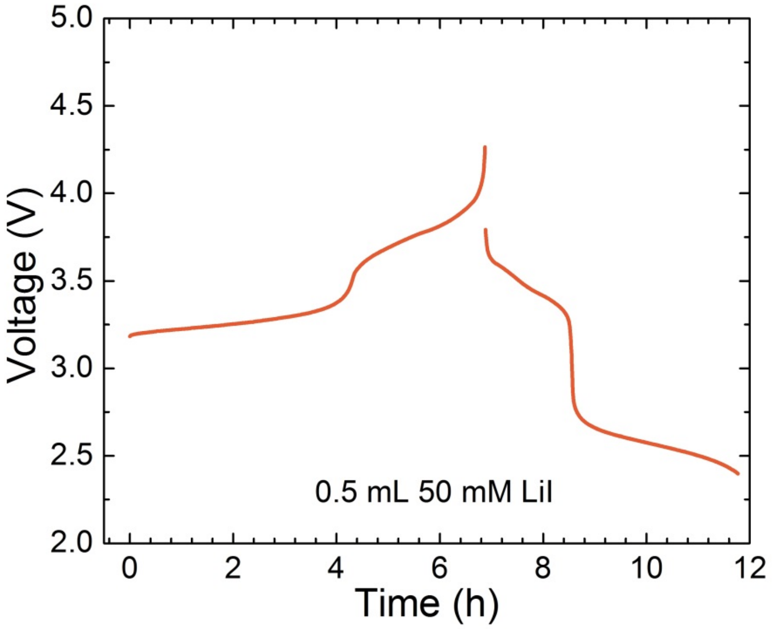

Supplementary Figure 11. The charge/discharge curve of a Li cell containing 0.5 mL catholyte consisting of 50 mM LiI in 0.5 M LiTFSI/DME. The cell has the same structure as the static Li-O_2 battery in Figure S7. The anolyte was 0.5 M LiTFSI in DEGDME. The test was conducted in Ar atmosphere. The current was 0.1 mA/cm^2. The effective ceramic area is 1 cm^2. The overall charging time is 6.8 h, nearly reaching the theoretical capacity of I^-/I_3^- and I_3^-/I_2. Therefore, the capacity from the redox mediators in static Li-O_2 batteries could be reliably estimated.

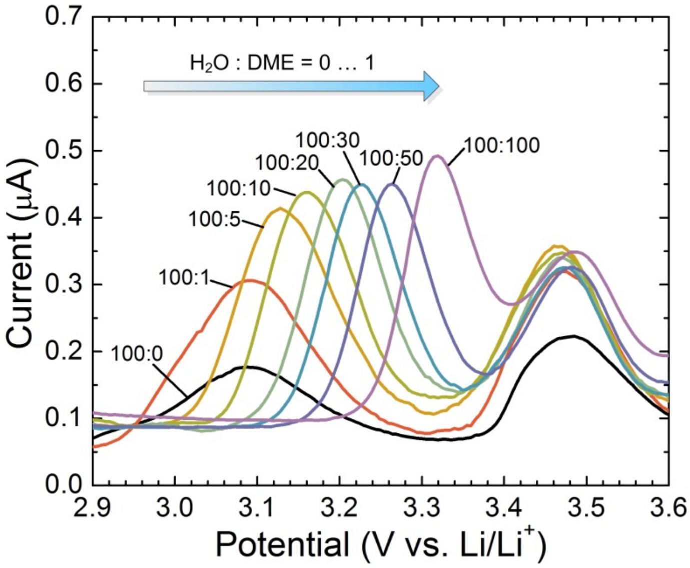

Supplementary Figure 12. The differential pulse voltammetry (DPV) measurements of 5 mM LiI in 0.5 M LiTFSI/DME electrolyte with different water contents. The working electrode was Pt disc electrode and the counter electrode was a piece of Pt plate. The reference electrode was $Ag/AgNO_3$. The potential is converted into Li/Li^+ scale for convenience. The potential range is from -0.4 to 0.3 V. The step potential is 0.005 V and the modulation amplitude is 0.025 V. Based on the DPV measurement, the redox potential of I^-/I_3^- has broadly a positive shift with the increase of water content in the aprotic electrolyte, while in contrast that of I_3^-/I_2 stays nearly constant. Precaution should be taken that as the $Ag/AgNO_3$ reference electrode was not calibrated when water content in the electrolyte bulk was increased, the changes of junction potential of the reference electrode may impose some error on the measurement.

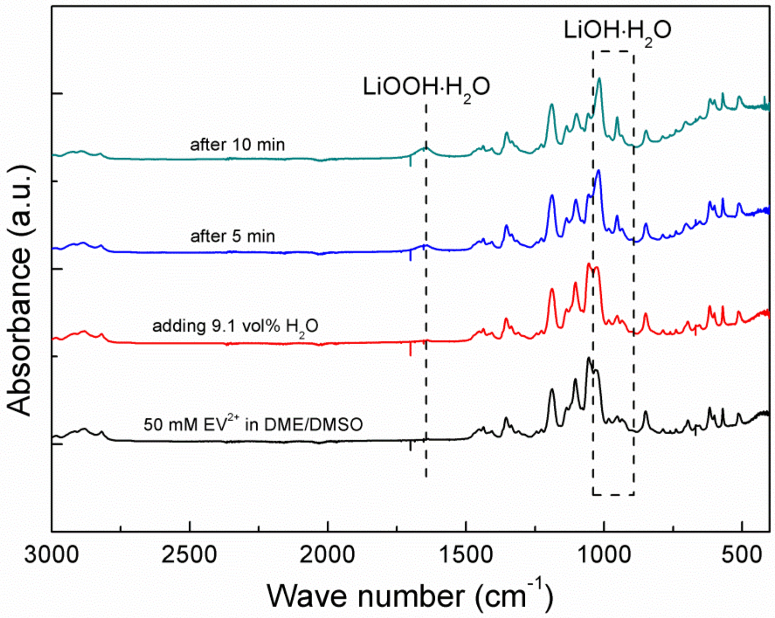

Supplementary Figure 13. ATR-FTIR spectra of the electrolyte and the ORR reaction products by EV^+ in the presence of 9.1 *vol.*% H_2O in the electrolyte.

To substantiate the presence of LiOH and LiOOH in the discharge (ORR) product, ATR-FTIR measurements were conducted. Here the ORR reaction was promoted by EV^+, which reduces O_2 in the presence of Li^+ and forms LiOH and LiOOH. As such, we firstly obtained EV^+ electrolyte by reducing 0.1 M EV^{2+} in 0.5 M LiTFSI / (DMSO:DME, 1:1) electrolyte with Li metal. 9.1 *vol.*% water was then added into the EV^+ electrolyte which was subsequently injected into a flow cell holder in O_2 atmosphere for ATR-FTIR measurement. As shown in Figure S13, the characteristic peaks of LiOH (910-1070 cm^{-1}, partly overlapped with that of the electrolyte) and $LiOOH{\cdot}H_2O$ (1641 cm^{-1}) appeared after 5 min reaction and became more pronounced after 10 min. This further verifies the formation of LiOH and LiOOH from the ORR reaction in the presence of 9.1 vol.% water in the electrolyte.

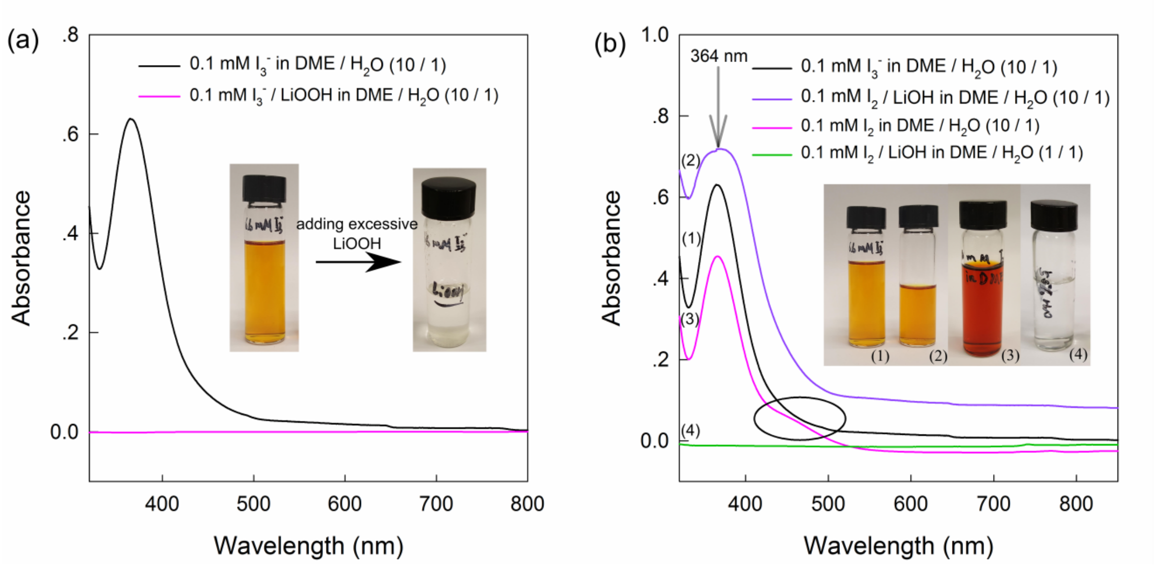

Supplementary Figure 14. (a) UV-Vis spectra of I_3^- in DME/H_2O (10/1) before and after adding excessive LiOOH. (b) UV-Vis spectra of standard 0.1 mM I_3^- solution and the reacted I_2 solution with LiOH. The inset of (b) shows the photos of 6.6 mM I_3^- (1), 10 mM reacted I_2 (2, DME/H_2O = 10/1), 10 mM I_2 (3), and 10 mM reacted I_2 (4, DME/H_2O = 1/1) solutions.

As the UV-Vis spectra shown in Figure S14a, the characteristic absorption peak of I_3^- at 364 nm (extended to the visible region) vanished after mixing with LiOOH·H_2O. As a result, the solution became nearly colourless. After adding excessive LiOH and stirring for 1 hour, the colour of I_2 solution in DME/H_2O (10:1) became slightly lighter (inset of Figure S14b). As the UV-Vis spectra shown in Figure S13b, after reacting with LiOH, the absorption of the I_2 solution became fairly identical to that of the standard solution of I_3^-. That is, the absorption at 400-500 nm was greatly attenuated with only the characteristic peak of I_3^- presented at around 364 nm, which confirms the existence of I_3^- in the reacted I_2 solution. The absorption peak broadening of I_2 solution after titration is presumably attributed to the scattering effect of small particles of LiOH, which has limited solubility in DME/H_2O (10:1).

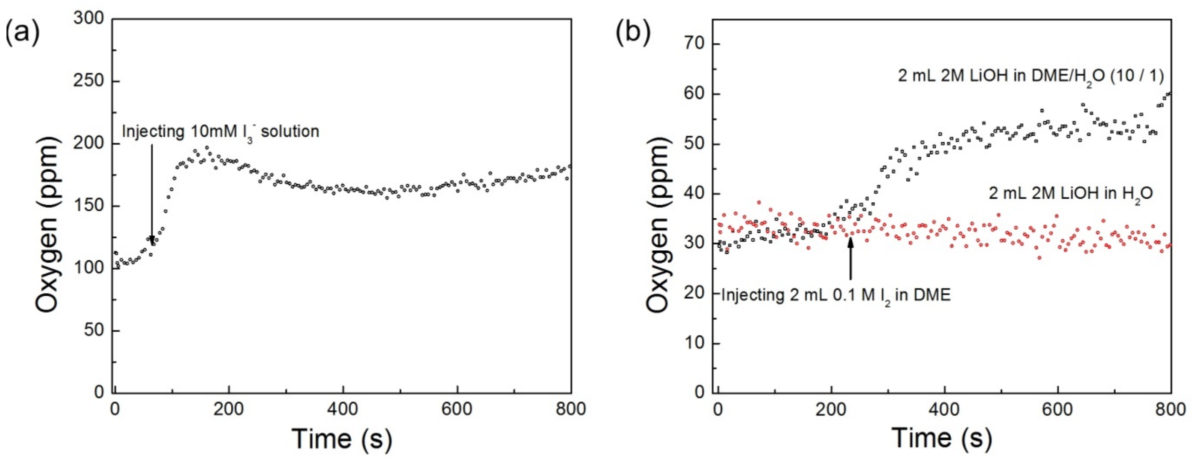

Supplementary Figure 15. Oxygen evolution recorded from the reactions of (a) LiOOH with I_3^- and (b) LiOH with I_2 monitored by mass spectrometry. The reactions of LiOH with I_2 were performed in solutions with different concentration of H_2O as labelled in the figure.

The mass spectrometric measurement in Figure S15a shows that O_2 evolves instantaneously upon mixing I_3^- with $LiOOH{\cdot}H_2O$. The mass spectrometric measurement of the reaction between LiOH and I_2 was conducted in two different solutions. As shown in Figure S15b, oxygen evolution was observed instantaneously after I_2 was injected into the 2 M LiOH suspension in DME/H_2O (10:1). Therefore, O_2 evolution is deemed part of the reaction between I_2 and LiOH in the DME/H_2O (10:1) electrolyte system, which is the same electrolyte used in the Li-O_2 cell. In contrast, no oxygen was detected when the solvent was changed to DME/H_2O (1:1).

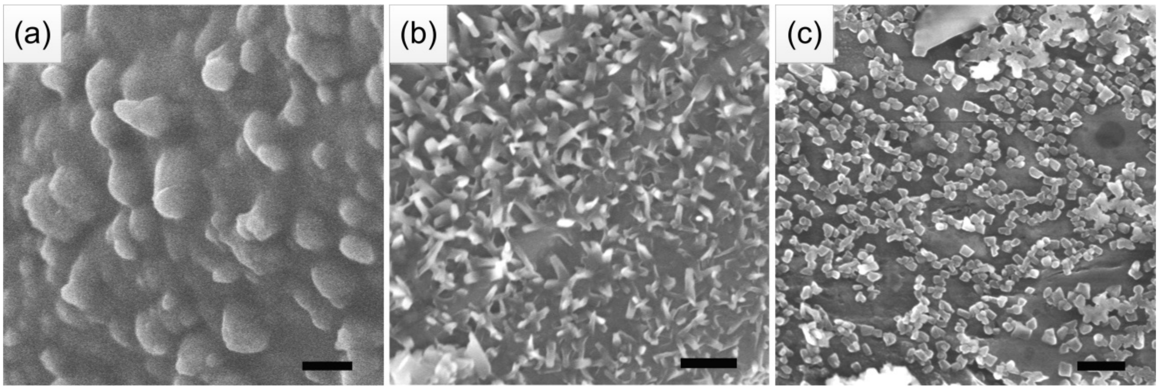

Supplementary Figure 16. Scanning electron microscopic images of the discharging products formed on the carbon felt of a static Li-O_2 battery in the case of (a) dry catholyte; (b) and (c) 9.1 *vol.*% H_2O in catholyte. Images (b) and (c) were taken on the different location of the same sample. The scale bar, 500 nm.

Crystal Structure of LiOOH·H_2O from Rietveld refinement of synchrotron XRD data

Space group: *P*-1

Lattice constants: *a* = 6.36610(4) Å, *b* = 6.08770(4) Å, *c* = 3.20629(2) Å,
α = 79.5953(4)° β = 101.8572(4)° γ = 102.3084(4)°

Cell volume = 117.591(2) Å^3 **Density**: 1.637 g/cm^3

Supplementary Table 1 *1Atomic coordinates and atomic displacement parameters*

**Name**	**X**	**Y**	**Z**	**U_i/U_e*100**	**Occupancy**	**bond valence sum**
**Li1**	0.66658	0.32573	0.70483	0.72	1.0	1.07
**O1W***	0.78412	0.20787	0.29499	2.27	1.0	2.09
**O2**	0.35481	0.23068	0.68065	1.72	1.0	0.92
**O3**	0.22354	0.34923	0.29743	1.72	1.0	1.07
**H1W***	0.72131	0.05463	0.26487	2.27	1.0	1.08
**H2W***	0.94145	0.18242	0.35354	2.27	1.0	0.90
**H3**	0.28696	0.27789	0.05970	1.82	1.0	1.02

** "W" in the atom name marks the O and H atoms that belong to water molecules*

R_{wp} = 7.86%, R_p= 6.1%, χ^2=2.93 Global instability index GII = 0.077

Crystal Structure of LiOOH·H_2O from DFT geometry optimisation

Space group: *P*-1

Lattice constants: *a* = 6.3178 Å, *b* = 6.1433 Å, *c* = 3.2120 Å,
α = 80.5404° β = 99.8294° γ = 103.3629°

Cell volume = 118.49 $Å^3$ **Density**: 1.625 g/cm^3

Supplementary Table 2 *Atomic coordinates and atomic displacement parameters*

Name	X	Y	Z	Occupancy
**Li1**	0.66707	0.32435	0.69873	1.0
**O1W**	0.78820	0.20761	0.28015	1.0
**O2**	0.34976	0.22548	0.68667	1.0
**O3**	0.22954	0.34311	0.31130	1.0
**H1W**	0.74272	0.03420	0.30581	1.0
**H2W**	0.95232	0.24676	0.30581	1.0
**H3**	0.27832	0.28934	0.04822	1.0

Supplementary References

1. Brooker MH, Bates JB. Raman and Infrared Spectral Studies of Anhydrous Li2CO3 and Na2CO3. *J. Chem. Phys.* **54**, 4788-4796 (1971).

2. Koura N*, et al.* Alkali carbonates: Raman spectroscopy, ab initio calculations, and structure. *J. Mol. Struct.* **382**, 163-169 (1996).

3. Freunberger SA*, et al.* Reactions in the rechargeable lithium–O2 battery with alkyl carbonate electrolytes. . *J. Am. Chem. Soc.* **133**, 8040-8047 (2011).

4. Parker SF, Refson K, Bewley RI, Dent G. Assignment of the vibrational spectra of lithium hydroxide monohydrate, LiOH·H2O. *J. Chem. Phys.* **134**, 084503 (2011).
